# Cognitive Training: A Field in Search of a Phenomenon

**DOI:** 10.1177/17456916221091830

**Published:** 2022-08-08

**Authors:** Fernand Gobet, Giovanni Sala

**Affiliations:** 1 Centre for Philosophy of Natural and Social Science, London School of Economics and Political Science; 2 Department of Epidemiology of Aging, National Center for Geriatrics and Gerontology, Obu, Japan; 3 Department of Psychology, University of Liverpool

**Keywords:** cognitive training, meta-analysis, methodology, working memory training

## Abstract

Considerable research has been carried out in the last two decades on the putative benefits of cognitive training on cognitive function and academic achievement. Recent meta-analyses summarizing the extent empirical evidence have resolved the apparent lack of consensus in the field and led to a crystal-clear conclusion: The overall effect of far transfer is null, and there is little to no true variability between the types of cognitive training. Despite these conclusions, the field has maintained an unrealistic optimism about the cognitive and academic benefits of cognitive training, as exemplified by a recent article (Green et al., 2019). We demonstrate that this optimism is due to the field neglecting the results of meta-analyses and largely ignoring the statistical explanation that apparent effects are due to a combination of sampling errors and other artifacts. We discuss recommendations for improving cognitive-training research, focusing on making results publicly available, using computer modeling, and understanding participants’ knowledge and strategies. Given that the available empirical evidence on cognitive training and other fields of research suggests that the likelihood of finding reliable and robust far-transfer effects is low, research efforts should be redirected to near transfer or other methods for improving cognition.

The last two decades have witnessed a considerable interest in cognitive training. Not only is cognitive training a multibillion-dollar industry (Ahuja, 2019), but its techniques are also used by large organizations, such as the U.S. military, and companies such as Cogmed, Lumosity, and Posit Science are often featured in the news. According to its proponents, cognitive training enhances children’s educational achievements, improves adults’ decision-making abilities, and alleviates the effects of aging on cognition. To try to support these claims, independent researchers and companies directly involved with cognitive training have conducted a substantial number of experiments.

The hypothesis that general cognitive abilities can be improved by cognitive-training tasks of fairly short duration is certainly counterintuitive to anyone familiar with the accumulated literature on intelligence and cognition. Considerable research indicates that fluid intelligence and working memory (WM) capacity cannot be improved through cognitive interventions (e.g., Deary, 2001; Shipstead et al., 2012). Likewise, substantial empirical evidence shows that learning and skill acquisition are domain-specific (e.g., Gobet, 2016; Sagi & Tanne, 1994; Simon & Chase, 1973). Showing positive effects of cognitive training would invalidate claims about the inflexibility of intelligence, WM capacity, learning, and expertise. There is no doubt that this would constitute a paradigm shift in psychology (Hurley, 2013), as is made clear by cognitive-training researchers. For example, Jaeggi et al. (2008) stated that “thus, in contrast to many previous studies, we conclude that it is possible to improve *Gf* without practicing the testing tasks themselves, opening a wide range of applications” (p. 6829), and Green and Bavelier (2003) concluded that “therefore, although video-game playing may seem to be rather mindless, it is capable of radically altering visual attentional processing” (p. 536).

An objective consideration of the evidence shows that these optimistic predictions have not been borne out by the data. The best way to evaluate the empirical evidence is to carry out meta-analyses, and we discuss the conclusions of several recent meta-analyses that covered WM training, video-game playing, chess playing, music, and exergame. Despite this contradicting evidence, researchers in the field maintain a high level of confidence that cognitive training is effective to improve general cognitive abilities, as exemplified recently by an article written by a group of 48 cognitive-training researchers (Green et al., 2019). Given that this article assembles many of the leading researchers in the field, we discuss it at some length in the second part of our article.

We argue that one of the reasons for this misplaced optimism is that the field, by and large, has ignored the role of study artifacts. We therefore spend a fair amount of space to communicate two critically important points mostly ignored in the literature about cognitive training. First, variability in the effect sizes obtained by different types of interventions does not necessarily imply that there are true differences between them: These differences might be simply due to the effects of sampling error and other kinds of artifacts. Second, before any conclusion can be reached about the variability of moderating variables, it is imperative to evaluate whether this variability is genuine (true heterogeneity) or due to random error.

## Defining Terms

Before diving into the details of our arguments, it is important to define key terms. *Cognitive training* refers to interventions using cognitive tasks or intellectually demanding activities, the goal of which is to enhance general cognitive ability (Sala & Gobet, 2017b, 2019). Thus, our definition includes not only “brain-training” tasks (i.e., tasks practicing basic cognitive abilities to enhance performance on other cognitive tasks, including everyday activities; Simons et al., 2016) but also activities such as music learning and video-game playing.^1^ This definition is fairly standard; for example, Strobach and Karbach’s (2016) book on cognitive training also includes a broader variety of activities than those covered by brain training, and so do numerous articles on the topic (Buschkuehl & Jaeggi, 2010; Katz et al., 2018; Simons et al., 2016; Taatgen, 2016).

The question of “transfer” is a central question in cognitive-training research. In line with the literature (e.g., Donovan et al., 1999), we define *near transfer* as the generalization of acquired skills across two (or more) domains that are closely related to each other (e.g., studying algebra to be better in geometry) and *far transfer* as the generalization of acquired skills across domains that are only loosely related to each other (e.g., studying algebra to improve in Chinese).^2^ Although this definition of transfer is qualitative and there are undoubtedly some ambiguous cases, in most cases, it is fairly easy to decide between near and far transfer. Everybody agrees that using a 3-back task after 2-back training is near transfer and that testing the effect of this training with an IQ test is far transfer. In addition, it is possible to use a more graded classification, such as “nearest transfer” (tasks that are the same as or similar to those used during training) and “less near transfer” (e.g., tasks that are different but still are aimed at improving performance in memory tasks; see Sala & Gobet, 2020b).

Our broad definition of cognitive training allows one to ask whether cognitive-training methods, taken as a group, provide broad cognitive and academic benefits (far transfer). We note that many researchers in the field would argue that this question is not legitimate. For example, Green et al. (2019) took as a starting point that “each individual type of behavioral intervention for cognitive enhancement (by definition) differs from all others in some way, and thus will generate different patterns of effects on various cognitive outcome measures” (p. 4). We believe that this hypothesis should be tested empirically rather than being accepted by fiat. In fact, as we show below, we have tested it and found that with respect to far transfer, it is incorrect.

## On the Importance of Sampling Error and Other Artifacts

In the first chapter of their book, Schmidt and Hunter (2015) presented a table summarizing the results of 30 studies on the link between job satisfaction and organizational commitment. They invited the reader to reach a conclusion about the strength of this link and about the variables that might moderate it and to draw implication for theory. The correlations ranged from –.10 to .56. Out of the 30 studies, 19 found a significant correlation, and 11 did not. Schmidt and Hunter discussed several patterns apparent in the data. For example, if only younger workers are considered, 19 out of the remaining 23 studies showed a significant correlation. Another pattern is that a significant correlation was found for 83% of the studies carried out in large organizations but only 50% in small organizations. Hence, the data seem to support the theory that organizational commitment grows over a 10-year period but then plateaus.

In fact, the data were generated by a Monte Carlo run in which the correlations were randomly sampled from a distribution with a population correlation of .33 and sample size was randomly selected from a distribution with a mean of 40. The organizational characteristics were allocated random values for each study. Therefore, the variation in the results were due to only chance (i.e., sampling error), and the large departures from the mean were obtained with small samples. According to Schmidt and Hunter (2015), this is a common situation in the psychological literature, and one should be aware that “‘conflicting results in the literature’ may be entirely artifactual” (p. 6). In addition, “many of the interactions hypothesized to account for differences in findings in different studies are nonexistent; that is, they are apparitions composed of the ectoplasm of sampling error and other artifacts” (p. 7).

It is our contention that by and large, the literature on cognitive training has underestimated the role of sampling error and other artifacts, which include issues with measurement, range restriction, and typographical errors, among others. Specifically, many researchers *assume* that distinct types of interventions will have different effects on far transfer—some interventions will have a positive effect, and others will not. But this is a hypothesis that researchers can test empirically while keeping in mind that the variability in results could be in reality artifactual. We tested this hypothesis in the meta-analyses and second-order meta-analysis that we discuss below and found that the hypothesis is incorrect empirically: The variability is artifactual. Thus, beyond random fluctuations, there are no differences between the different types of intervention: Their effect on far-transfer tasks is null when sampling error, publication bias, and type of control group are taken into account. We get the same results when meta-analyses are carried out within one domain (e.g., action video games vs. nonaction video games) or between domains (i.e., the second-order meta-analysis comparing the effects of WM training, video-game playing, etc.). Thus, rather than limiting researchers to piecemeal conclusions (e.g., Intervention 1 does not lead to far transfer; Intervention 2 does not lead to far transfer), we show that it is possible to reach a conclusion that applies to the broad category of cognitive training. Reaching broad generalizations supported by empirical evidence is the hallmark of scientific progress (Braithwaite, 1960; Chow, 1987).

We give this preview of our results because the importance of sampling error and other artifacts has been systematically overlooked in the cognitive-training field. Assuming that different treatments lead to different effects was a plausible hypothesis at the beginning of the research, but it is not anymore. However, the field has, on the whole, clung to this hypothesis, and many of the points we discuss next hinge on the failure to recognize the role played by sampling error.

## Meta-Analytic Evidence

### The rationale behind meta-analysis

Disagreements often occur in quantitative empirical research, and meta-analysis is considered one of the most effective tools for resolving them. Meta-analysis offers a set of statistical methods for integrating research findings on a particular topic across studies (Borenstein et al., 2009; Schmidt & Hunter, 2015). It has three main objectives: (a) to estimate the magnitude of an overall effect and its confidence intervals, (b) to quantify the consistency of the literature (i.e., whether there is variability in the findings across studies), and (c) to reveal the role of potential moderators.

The overall effect size is calculated by averaging the effect sizes (e.g., standardized mean differences between two groups) obtained from the primary studies. Each effect size is weighted on precision (i.e., inverse of the sampling error variance),^3^ which is primarily, sometimes solely, a function of sample size. The larger the sample, the bigger the weight of the effect in the analysis will be.

An essential piece of information offered by meta-analysis is the degree of between-studies true variance (τ^2^). In brief, the variance observed in any population of effect sizes can be decomposed, at the very least, into true variance and artifactual variance (e.g., variance because of sampling error and measurement error). Whereas the former warrants an explanation, the latter does not. Specifically, τ^2^ estimates the between-studies variance in the population of the effect sizes that is not due to sampling error. A low or null τ^2^ suggests that no moderating variable affects the magnitude of the effects across the primary studies. If τ^2^ ≈ 0, then it can be inferred that there is only one true effect in the literature. The accuracy of this overall effect is provided by its standard error, which is a function of the number of observations included in the meta-analytic model. By contrast, a high τ^2^ indicates that the magnitude of the effect is moderated by some variables (e.g., type of control group). Accounting for between-studies true variance, when it exists, is fundamental to providing reliable and interpretable meta-analytic estimates.

Note that unless one has strong a priori predictions about the type of moderators that might play a role, it is necessary to first test whether there is true heterogeneity in the data. If this is not the case, then no moderator analysis should be carried out to not capitalize on sampling error (Schmidt & Hunter, 2015). If there is true heterogeneity, one should test whether specific moderators are statistically significant. Only in this case is it appropriate to carry out a detailed moderator analysis. A final caveat is that testing a large number of potential moderators is inappropriate because this capitalizes on chance (Type I error).

### What do meta-analyses tell researchers about cognitive training?

As noted above, we have carried out several meta-analyses about cognitive training.^4^ We have repeatedly found that the true far-transfer effect size, when estimated from the comparison of treatment versus active control group, is close to zero. This outcome has been found for WM training (Aksayli et al., 2019; Sala, Aksayli, Tatlidil, Gondo, & Gobet, 2019; Sala & Gobet, 2020b), video-game playing (Sala et al., 2018), exergames (Sala et al., 2021), and music training (Sala & Gobet, 2017c, 2020a, 2020b). The exception is chess (Sala & Gobet, 2016), for which too few studies with an active control group have been carried out; however, the few available studies with an active control group suggest a lack of far transfer (e.g., Sala & Gobet, 2017a).

These meta-analyses were carried out with different methods. Sala, Aksayli, Tatlidil, Tatsumi, et al. (2019) redid them with the same method. Table 1 presents a summary of these meta-analyses and the results of adjustments enabled by second-order meta-analyses (see the following section) for the experimental results not corrected for publication bias and including both active and passive control groups. Table 2 presents the corresponding meta-analyses when the studies are corrected for publication bias and include only active control groups—a better estimate of the true effect of cognitive training. As we show, the estimated effect sizes of the first-order meta-analyses are small in Table 1 (range = 0.04–0.19) and essentially zero in Table 2 (range = −0.03 to 0.02). In both tables, the amount of true heterogeneity is very small.

**Table 1.** First- and Second-Order Meta-Analyses With the Uncorrected (Naive) Overall Effect Sizes, Far Transfer Only

Population	*k*_i_	${\bar{g}}_{i}$	$S_{gi}^{2}$	$\tau^{2}$	$\mathrm{Adjusted}\;{\bar{g}}_{i}$
First-order meta-analyses summary					
WM (TD children)	25	0.13	0.060	0.006	0.12
WM (LD children)	18	0.12	0.032	0.002	0.12
WM (adults)	44	0.12	0.041	0.003	0.12
WM (older adults)	32	0.13	0.085	0.035	0.12
Action VG (adults)	32	0.08	0.073	0.000	0.12
Nonaction VG (adults)	16	0.15	0.047	0.012	0.12
VG (older adults)	10	0.04	0.033	0.000	0.12
Music (TD children)	36	0.19	0.087	0.042	0.12
Chess (TD children)	9	0.13	0.049	0.031	0.12
Exergames (older adults)	11	0.15	0.079	0.021	0.12
Second-order meta-analysis summary results $\overset{=}{g}=\;$ 0.12 (second-order grand mean) $\sigma_{e}^{2}\;=0.00235$ (second-order sampling-error variance) $\sigma_{{\bar{g}}_{i}}^{2}=0.00129$ (observed between-first-order-meta-analyses variance) $\sigma^{2}\;=\;0$ (true between-first-order-meta-analyses variance)					

Note: Data From Sala, Aksayli, Tatlidil, Tatsumi, et al. (2019). *k*_i_ $k_{i}$ = number of samples; ${\bar{g}}_{i}=\;$ first-order overall effect size; $S_{gi}^{2}$ = variance of the observed *g*s; $\tau^{2}=\;$ amount of true heterogeneity; adjusted ${\bar{g}}_{i}\;$ = adjusted first-order overall effect size; TD = typically developing; LD = learning disabilities; VG = video games; WM = working memory.

**Table 2.** First- and Second-Order Meta-Analyses With the Corrected Overall Effect Sizes (Only Active Control Groups), Far Transfer Only

Population	$k_{i}$	${\bar{g}}_{i}$	$S_{gi}^{2}$	$\tau^{2}$	$\mathrm{Adjusted}\;{\bar{g}}_{i}$
First-order meta-analyses summary					
WM (TD children)	15	0.01	0.064	0.000	0.00
WM (LD children)	12	0.02	0.111	0.000	0.00
WM (adults)	27	0.00	0.213	0.000	0.00
WM (older adults)	16	0.01	0.009	0.000	0.00
Action VG (adults)	34	−0.01	0.107	0.011	0.00
Nonaction VG (adults)	6	0.00	0.033	0.000	0.00
VG (older adults)	4	−0.03	0.033	0.000	0.00
Music (TD children)	17	−0.02	0.055	0.012	0.00
Chess (TD children)	3	0.01	0.032	0.000	0.00
Exergames (older adults)	8	−0.02	0.072	0.000	0.00
Second-order meta-analysis summary results $\overset{=}{g}$ = 0.00 (second-order grand mean) $\sigma_{e}^{2}\;=0.00302$ (second-order sampling-error variance) $\sigma_{{\bar{g}}_{i}}^{2}\;=0.00014$ (observed between-first-order-meta-analyses variance) $\sigma^{2}\;=0$ (true between-first-order-meta-analyses variance)					

Note: Data From Sala, Aksayli, Tatlidil, Tatsumi, et al. (2019). *ki* = number of samples; ${\bar{g}}_{i}=\;$ first-order overall effect size; $S_{gi}^{2}$ = variance of the observed *g*s; $\tau^{2}\;$ = amount of true heterogeneity; $\mathrm{adjusted}\;{\bar{g}}_{i}\;$ = adjusted first-order overall effect size; TD = typically developing; LD = learning disabilities; VG = video games; WM = working memory.

Thus, the meta-analyses allowed us to quantify, with respect to far-transfer effects, the extent to which the literature is mixed and could explain any between-studies true variance. An important conclusion was that the results are *not* inconsistent and thus do not depend on differences in methodologies between researchers. That is, once baseline differences were controlled for, the only appreciable source of true variance (which is often quite low) is the type of control group. In other words, the debate about the literature being mixed and the results inconsistent is just much ado about nothing. Far-transfer effects do not exist. Cognitive-training researchers seem to incorrectly equate sampling-error variance and true variance: Terms such as “τ^2^,” “true variance,” or “true heterogeneity” rarely appear in cognitive-training reviews. In addition, it seems that cognitive-training researchers fail to understand that it is absolutely normal that significantly positive effects are sometimes found (e.g., when comparing treatment groups with active control groups on far-transfer measures) even if the true effect is zero. Specifically, by chance, we expect a portion (5%) of the measurements to be statistically significant (*p* < .05, one-tailed). Effect sizes in a given literature are mathematically bound to differ because of sampling error. Variability across and within the studies is the rule, not the exception.

### A step further: second-order meta-analysis

Second-order meta-analysis is a procedure designed by Schmidt and Oh (2013) for integrating findings of first-order (i.e., conventional) meta-analyses. This technique estimates a grand mean of the first-order overall effect sizes and, most notably, the between-meta-analyses true variance. Second-order meta-analysis represents the current highest level of cumulative knowledge in quantitative research.

In Sala, Aksayli, Tatlidil, Tatsumi, et al. (2019), we applied second-order meta-analysis to cognitive-training data (for results about far transfer, see Tables 1 and 2). The analysis included 14 statistically independent first-order meta-analyses (332 samples, 1,555 effect sizes, and 21,968 participants) of near- and far-transfer effects in different populations (e.g., children, adults, and older adults). As shown in Tables 1 and 2, the training programs covered were WM training, action- and nonaction-video-game training, music training, chess training, and exergame training. The key results were as follows. First, near transfer occurs even when placebo effects are controlled for and seems to be moderated by the age of the participants. Second, far transfer is negligible (uncorrected overall effect) or null (when placebo effects and publication bias are ruled out). Third, within-studies (ω^2^) and between-studies true variance (τ^2^) are small to null with far transfer. Fourth, second-order sampling error (i.e., the residual sampling error from first-order meta-analyses) explains all the between-meta-analyses variance with far transfer. That is, we found no evidence of either within-studies, between-studies, or between-meta-analyses true variance. These results strongly corroborate the idea that although near transfer is real and the magnitude of its effect is moderated by the population examined, the observed far transfer is due to factors that are unspecific (i.e., it occurs regardless of the type of training regimen or population), such as placebos. (This conclusion is buttressed by the results of Kassai et al., 2019, who carried out a meta-analysis on training components of children’s executive-functions skills, a type of training not covered by our second-order meta-analysis.)

### Other cognitive-training programs

For some cognitive-training programs, there are not enough studies to perform a proper meta-analysis. Examples include the ACTIVE trial, commercial brain-training games (e.g., Neuroracer, Lumosity, and BrainHQ), and multidomain training programs (Binder et al., 2016; Buitenweg et al., 2017; Duyck & Op de Beeck, 2019). To date, none of these regimens have shown compelling evidence, or any evidence at all, of training-induced far transfer to either cognitive tests or real-life skills (for reviews, see Sala & Gobet, 2019; Simons et al., 2016). These studies are thus in line with the findings reviewed above.

### Active versus passive control groups

Recently, Au et al. (2020) questioned the use of active control groups as currently used in the cognitive-training literature. These authors carried out a meta-analysis and a meta-meta-analysis on the effects of cognitive interventions, focusing on the differences between passive and active control groups. They took their results as showing that there is no meaningful performance difference between the two types of control groups. This is clearly different from the conclusions obtained in our meta-analyses with respect to far transfer. Why did they obtain different results? We believe that these differences result from several suboptimal (to incorrect) decisions made by Au et al.

Most importantly, the meta-meta-analysis was performed in a less than optimal way. Statistically dependent meta-analyses—that is, meta-analyses including the same primary studies—were put together in the same model.^5^ This procedure violates the assumption of independence. This often leads to underestimating sampling error variance and, hence, overestimating true variance, which results in errors in calculating effect sizes and confidence intervals (Schmidt & Hunter, 2015; Schmidt & Oh, 2013). In addition, only meta-analyses published until 2016 were included, which has the consequence of ignoring a substantial amount of evidence. Finally, Au et al. (2020) mixed different types of information: (a) different types of training, including cognitive-training interventions, mnemonics (Floyd & Scogin, 1997; Verhaeghen et al., 1992), and serious games (Wouters et al., 2013), and (b) near-transfer (e.g., Uttal et al., 2013) and far-transfer (e.g., Lampit et al., 2014) outcomes (there is little to no placebo effect in near transfer in our meta-analyses, too). In conclusion, Au et al.’s results do not represent any compelling evidence that the choice of control group (passive or active) is irrelevant to the results in the cognitive-training literature.

Technical issues aside, the most relevant aspect of the problem is defining what qualifies as an active control group. Simons et al. (2016) highlighted that active controls should be designed to isolate the variable of interest (i.e., the effect of the training program) as accurately as possible. This means that to rule out placebo effects, active control groups should be engaged in activities that are cognitively demanding and trigger positive expectations on their effectiveness in the participants (Boot et al., 2013). Therefore, control activities should differ from the cognitive-training program regarding only the key element that is hypothesized to enhance the target cognitive skill or skills. For example, the far-transfer effects of WM training regimens could be tested by employing adaptive visual-search tasks (e.g., Guye & von Bastian, 2017; Hering et al., 2017). Although cognitively demanding and perceived as effective training, these tasks lack the “WM training component.” Using nonadaptive WM training tasks is, in our opinion, a slightly less desirable choice.

Meta-analyses and reviews about cognitive training often do not apply Simons et al.’s (2016) criterion for defining a control activity as active (e.g., Au et al., 2020; Teixeira-Santos et al., 2019). Rather, control groups engaged in any alternative activity (e.g., non-cognitively demanding filler tasks) are considered as active. This less stringent (suboptimal) criterion is another source of discrepancy between meta-analyses in the literature.

Finally, note that our meta-analyses do not show that placebo effects occur in all cognitive-training programs. For example, they are not present in either action- or nonaction-video-game training (Sala et al., 2018). However, we did find that placebos always occur in WM training when it comes to far transfer (Sala & Gobet, 2020b). These placebos are around 0.15 to 0.20 standardized mean difference at best and often affected by publication bias.

### Publication bias and laboratory bias

In our second-order meta-analysis, we estimated a small publication-bias effect (0.05–0.10 standardized mean differences). Publication bias thus seems to be a minor issue in the cognitive-training literature. In fact, this finding appears to be in line with the current state of the art in psychology (Stanley et al., 2018). Of more interest are probably the anomalous effects reported by two laboratories involved in cognitive-training studies, effects that were identified by meta-analyses (Bediou et al., 2018; Sala, Aksayli, Tatlidil, Gondo, & Gobet, 2019). The effect sizes reported by these laboratories, which are unusually large compared with those found by other laboratories, are a nonnegligible source of variability in the cognitive-training literature, and an important task for further research will be to understand the reason for these discrepancies.

First, the Padua laboratory (Borella and colleagues) has carried out more than 10 studies implementing a particular WM training regimen in older adults (Categorization Working Memory Span [CWMS] task; for more details, see Borella et al., 2017). In nearly all of these studies, medium to large effect sizes were found in both near- and far-transfer measures. The other studies in the field that used the CWMS task reported small to null overall effect sizes (Sala, Aksayli, Tatlidil, Gondo, & Gobet, 2019). This marked difference between the findings of the Padua laboratory and the ones reported by other laboratories is probably due to the peculiar type of active control group employed by the former. Rather than a cognitively demanding activity, the control subjects were often asked to fill in biographical questionnaires. This type of filler task does not meet the standards of an active task. A study that employed the CWMS training regimen and compared its effects against a cognitively active control task (adaptive visual-search training) found small near-transfer effects and no far-transfer effect (Hering et al., 2017).

Second, Green and Bavelier’s studies about the benefits of playing action video games reported much greater effects than all the other studies in the field (Bediou et al., 2018). This anomaly—which is captured in the asymmetry of the distribution of the effect sizes—is, in all probability, due to the fact that some effect sizes were suppressed from the primary studies (Bavelier’s personal communication reported in Boot et al., 2011) or have been incorrectly reported as coming from different samples. These issues have been documented in several articles by Simons and Boot (Boot et al., 2011; Hilgard et al., 2019) and have led to a series of corrections of Green and Bavelier’s findings (e.g., Green & Bavelier, 2019, 2020).

### Between-individuals differences in far transfer

A common argument against meta-analytic evidence is that it does not account for within-studies individual differences. In a very general sense, this argument is correct. Meta-analysis does not provide any detailed information regarding within-studies, between-subjects differences. Meta-analysis is designed for estimating the magnitude and consistency of overall effects. Nonetheless, this does not mean that meta-analytic evidence is unreliable. In fact, the combination of null overall far-transfer effects and null between-studies true variability suggests that between-individuals, within-studies differences seem to matter very little in cognitive training. That being said, we think that it is useful to discuss how some authors come to the conclusion that individual differences do show up in cognitive-training data despite a lack of clear-cut effects.

Jaeggi et al. (2011) presented the argument that there are between-individuals differences in far transfer (even if the mean difference between trainees and control subjects is close to zero) because there is a correlation between gains in the trained task and gains in the transfer tasks in the experimental group. The idea is that the more one improves on the training task (e.g., *n*-back), the more one benefits from the training in terms of far transfer (e.g., improvement in the Raven’s matrices).

This argument is incorrect statistically. Positive correlations between gains occur every time within-sessions (i.e., same time point) covariances are bigger than between-sessions covariances. However, there is no good reason why this should be considered as evidence in favor of a training effect (for all the details, see Tidwell et al., 2014).

Another common incorrect argument relies on the negative correlation occurring between far-transfer pretest scores and pretest/posttest gains. This correlation is sometimes presented as evidence of an individual-based compensatory effect (e.g., Karbach et al., 2015). Put simply, a given cognitive-training regimen is believed to be particularly effective for individuals who performed poorly at baseline assessment (i.e., Subject × Treatment interaction). However, such negative correlations are likely to be, at least in part, statistical artifacts due to regression to the mean (Smoleń et al., 2018). Therefore, correlations between pretest/posttest gains and pretest scores alone cannot be considered as evidence for true individual differences in training-induced transfer effects.

Beyond the above statistically incorrect inferences, we note that postulating between-individuals differences when the overall far-transfer effect is zero leads to absurd conclusions, especially if no true between- or within-studies variance is observed. In fact, if a subgroup of participants outperforms the control participants (true positive effect size), that means that the other subgroup is outperformed by the control participants (true negative effect size) because the mean effect is zero. Now, why should cognitive-training programs exert a true negative effect (i.e., damage) on cognition? It is obvious that if the overall effect is zero, then the training has no impact on one’s domain-general cognitive skills regardless of any covariate. On the other hand, if researchers assume that the training is effective (i.e., true positive effect size) for a subgroup of individuals and ineffective yet not detrimental (i.e., true null effect size) for the other group, then they would observe an attenuated but still positive overall effect size. This scenario is, however, inconsistent with the empirical data (the observed overall effect is zero).

Finally, the above correlation-based arguments seem odd. It is well known that correlations do not constitute any evidence of causality. Only the inclusion of a control group can isolate the variable of interest (i.e., training-induced far-transfer effects). For example, Smoleń et al. (2018) showed that modeling correlation with structural models may, in principle, provide some evidence of a true compensatory effect (i.e., beyond regression to the mean). However, it is necessary to include a control group to demonstrate that such an effect is *caused* by training programs. More prosaically, it is unclear why time and resources should be invested to enroll an entire control group if correlations were enough to establish a causality link between a person’s performance in training tasks and cognitive enhancement. We must conclude that, in the current state of the art, appealing to putative individual differences in cognitive training appears more like an attempt to make far-transfer null effects worth some optimism and further research rather than a proper scientific hypothesis.

## What Is Wrong With the Cognitive-Training Hypothesis?

As is clear from the empirical evidence reviewed in the previous sections, the likelihood that cognitive training provides broad cognitive and academic benefits is very low indeed; therefore, resources should be devoted to other scientific questions—it is not rational to invest considerable sums of money on a scientific question that has been essentially answered by the negative. In a recent article, Green et al. (2019) took the exact opposite of this decision—they strongly recommended that funding agencies should increase funding for cognitive training. This obviously calls for comments.

The aim of Green et al.’s (2019) article was to provide methodological recommendations and a set of best practices for research on the effect of behavioral interventions aimed at cognitive improvement. Among others, the addressed issues include the importance of distinguishing between different types of studies (feasibility, mechanistic, efficacy, and effectiveness studies), the type of control groups used, and expectation effects. Many of the points addressed in detail by Green et al. reflected sound and well-known research practices (e.g., necessity of running studies with sufficient statistical power, need for defining the terminology used, and importance of replications; see also Simons et al., 2016).

However, the authors made disputable decisions concerning central questions. These include whether superordinate terms such as “cognitive training” and “brain training” should be defined, whether a discussion of methods is legitimate while ignoring the empirical evidence for or against the existence of a phenomenon, the extent to which meta-analyses can compare studies obtained with different methodologies and cognitive-enhancement methods, and whether multiple measures should be used for a latent construct such as intelligence.

### Lack of definitions

Although Green et al. (2019) emphasized that “imprecise terminology can easily lead to imprecise understanding and open the possibility for criticism of the field,” they opted to not provide an explicit definition of “cognitive training” (p. 4). Nor did they define the phrase “behavioral interventions for cognitive enhancement,” used throughout their article. Because they specifically excluded activities such as video-game playing and music (p. 3), we surmised that they used “cognitive training” to refer to computer tasks and games that aim to improve or maintain cognitive abilities such as WM. The term “brain training” is sometimes used to describe these activities, although it should be mentioned that Green et al. objected to the use of the term.

Note that researchers investigating the effects of activities implicitly or explicitly excluded by Green et al. (2019) have emphasized that the aim of those activities is to improve cognitive abilities and/or academic achievement, for example, chess (Jerrim et al., 2017; Sala et al., 2015), music (Gordon et al., 2015; Schellenberg, 2006), and video-game playing (Bediou et al., 2018; Feng et al., 2007). For example, Gordon et al.’s (2015) abstract concluded by stating that “results are discussed in the context of emerging findings that music training may enhance literacy development via changes in brain mechanisms that support both music and language cognition” (p. 1).

Green et al. (2019) provided a rationale for not providing a definition. Referring to “brain training,” they wrote:

We argue that such a superordinate category label is not a useful level of description or analysis. Each individual type of behavioral intervention for cognitive enhancement (by definition) differs from all others in some way, and thus will generate different patterns of effects on various cognitive outcome measures. (p. 4)

They also noted that even using subcategories such as “working-memory training” is questionable. They did note that “there is certainly room for debate” (p. 4) about whether to focus on each unique type of intervention or to group interventions into categories.

In line with common practice (e.g., De Groot, 1969; Elmes et al., 1992; Pedhazur & Schmelkin, 1991), we take the view that definitions are important in science. Therefore, in this article, we have proposed a definition of “cognitive training” (see “Defining Terms” section above), which we have used consistently in our research.

### Current state of knowledge and meta-analyses

A sound discussion of methodology in a field depends on the current state of knowledge in this field. Whereas Green et al. (2019) used information gleaned from previous and current cognitive-training research to recommend best practices (e.g., use of previous studies to estimate the sample size needed for well-powered experiments), they also explicitly stated that they will not discuss previous controversies. We believe that this is a mistake because, as just noted, the choice of methods is conditional on the current state of knowledge. In our case, a crucial ingredient of this state is whether cognitive-training interventions are successful—specifically, whether they lead to far transfer. One of the main “controversies” precisely concerns this question, and thus it is unwise to ignore it.

Green et al. (2019) were critical of meta-analyses and argued that studies cannot be compared:

For example, on the basic research side, the absence of clear methodological standards has made it difficult-to-impossible to easily and directly compare results across studies (either via side-by-side contrasts or in broader meta-analyses). This limits the field’s ability to determine what techniques or approaches have shown positive outcomes, as well as to delineate the exact nature of any positive effects – e.g., training effects, transfer effects, retention of learning, etc. (p. 3)

These comments wholly underestimate what can be concluded from meta-analyses. Like many other researchers in the field, Green et al. (2019) assumed that (a) the literature is mixed and, consequently, (b) the inconsistent results depend on differences in methodologies between researchers. However, *assuming* that there is *some* between-studies inconsistency and speculating on where this inconsistency stems from is not scientifically apposite (see “The Importance of Sampling Error and Other Artifacts” section above). Rather, *quantifying* the between-studies true variance (τ^2^) should be the first step to take.

### Using latent factors

In the section “Future Issues to Consider With Regard to Assessments,” Green et al. (2019, pp. 16–17) raised several issues with using multiple measures for a given construct such as WM. This practice has been recommended by authors such as Engle et al. (1999) to reduce measurement error. Several of Green et al.’s arguments merit discussion.

A first argument is that using latent factors—as in confirmatory factor analysis—might hinder the analysis of more specific effects. This argument is incorrect because the relevant information is still available to researchers (see Kline, 2016; Loehlin, 2004; Tabachnik & Fidell, 1996). By inspecting factor loadings, one can examine whether the preassessment/postassessment changes (if any) affect the latent factor or only specific tests (this is a longitudinal-measurement-invariance problem). Green et al. (2019) seemed to equate multi-indicator composites (e.g., summing *z* scores) with latent factors. Composite measures are the result of averaging or summing across a number of observed variables and cannot tell much about any task-specific effect. A latent factor is a mathematical construct derived from a covariance matrix within a structural model that includes a set of parameters that links the latent factor to the observed variables. That being said, using multi-indicator composites would be an improvement compared with the current standards in the field.

A second argument is that large batteries of tests induce motivational and/or cognitive fatigue in participants, especially with particular populations. Although this may be true, for example with older participants, large batteries have been used in several cognitive-training studies, and participants were able to undergo a large variety of testing (e.g., Guye & von Bastian, 2017). Nevertheless, instead of assessing many different constructs, it may be preferable to focus on one or two constructs at a time (e.g., fluid intelligence and WM). Such a practice would help reduce the number of tasks and the amount of fatigue.

Another argument concerns carryover and learning effects. The standard solution is to randomize the presentation order of the tasks. This procedure, which ensures that bias gets close to zero as the number of participants increases, is generally efficient if there is no reason to expect an interaction between treatment and order (Elmes et al., 1992). If this is the case, another approach can be used: counterbalancing the order of the tasks. However, complete counterbalancing is difficult with large numbers of tasks, and in this case, one often has to be content with incomplete counterbalancing using a Latin square (for a detailed discussion, see Winer, 1962).

A final point made by Green et al. (2019) is that using large batteries of tasks increases the rate of Type I errors. Although this point is correct, it is not an argument against multi-indicator latent factors. Rather, it is an argument in favor because those do not suffer from this bias. In addition, latent factors aside, there are many methods designed for correcting α (i.e., the significance threshold) for multiple comparisons (e.g., Bonferroni, Holm, false-discovery rate). Increased Type I error rates are a concern with researchers who ignore the problem and do not apply any correction.

One reasonable argument is that latent factor analysis requires large numbers of participants. The solution is offered by multilab trials. The ACTIVE trial—the largest experiment carried out in the field of cognitive training—was, indeed, a multisite study (Rebok et al., 2014). Another multisite cognitive-training experiment is currently ongoing (Mathan, 2018).

To conclude this section, we emphasize two points. First, it is well known that in general, single tests possess low reliability. Second, multiple measures are needed to understand whether improvements occur at the level of the test (e.g., *n*-back) or at the level of the construct (e.g., WM).

### Some methodological recommendations

We are not as naive as to believe that our analysis will deter researchers in the field to carry out much more research on the putative far-transfer benefits of cognitive training despite the lack of any empirical evidence. We thus provide some advice about the directions that should be taken so that not all resources are spent in search of a chimera.

#### Making methods and results accessible, piecemeal publication, and objective report of results

We broadly agree with the methodological recommendations made by Green et al. (2019), such as reporting not only *p* values but also effect sizes and confidence intervals, and the need for well-powered studies. We add a few important recommendations (for a summary of the recommendations throughout this article, see Table 3). To begin with, it is imperative to put the data, analysis code, and other relevant information online. In addition to providing supplementary backup, this allows other researchers to closely replicate the studies and to carry out additional analyses (including meta-analyses)—important requirements in scientific research. By the same token and in the spirit of Open Science, researchers should reply to requests from meta-analysts asking for summary data and/or the original data. In our experience, response rate is currently 20% to 30% at best (e.g., Sala et al., 2018). Although we understand that it may be difficult to answer such replies positively when data were collected 20 years or more ago, there is no excuse for data collected more recently.

**Table 3.** Key Recommendations for Researchers

General recommendations
Provide precise definitions of key terms (e.g., cognitive training, active control group, near and far transfer).
Avoid piecemeal publication; when this is unavoidable, provide references to the articles sharing the results.
Avoid hyperbole and incorrect generalization.
Use well-specified theories (e.g., computational models) to derive predictions about the potential effectiveness of cognitive training.
Use detailed measures (e.g., eye movements, mouse clicks) to understand the detail of the cognitive mechanisms mediating potential cognitive transfer.
Understand the strategies used by the participants.
Test interventions in silico before testing them in vivo.
Carry out a task analysis of the tasks used in pretest and posttest as well as in training.
Focus on near transfer because far transfer is elusive.
Recommendations about statistics and data curation
Put the data, analysis code, and other relevant information online.
Report results correctly and objectively; do not capitalize on chance with suspect statistical practices.
Reply to requests from meta-analysts asking for summary data and/or the original data.
When estimating latent factors, use multiple measures for each factor.
Randomize the presentation order of the tasks.
Use meta-analytic evidence for assessing the plausibility of cognitive-training interventions.
Pay attention to true heterogeneity in the data for making informed conclusions.

Just like other questionable research practices, piecemeal publication should be avoided (Hilgard et al., 2019). If dividing the results of a study into several articles cannot be avoided, the articles should clearly and unambiguously indicate the fact that this has been done and should reference the articles sharing the results.

There is one point made by Green et al. (2019) with which we wholeheartedly agree: the necessity of reporting results correctly and objectively without hyperbole and incorrect generalization. The field of cognitive training is littered with exaggerations and overinterpretations of results (see Simons et al., 2016). A fairly common practice is to focus on the odd statistically significant result even though most of the tests turn out nonsignificant. This is obviously capitalizing on chance and should be avoided at all costs.

In a similar vein, there is a tendency to overinterpret results of studies using neuroscience methods. A striking example was recently offered by Schellenberg (2019), who showed that in a sample of 114 journal articles published in the last 20 years on the effects of music training, causal inferences were often made although the data were only correlational; neuroscientists committed this logical fallacy more often than psychologists. There was also a rigid focus on learning and the environment and a concurrent neglect of alternative explanations, such as innate differences. Another example consists in inferring far transfer when neuroimaging effects are found but not behavioral effects. However, such an inference is illegitimate.

#### The need for detailed analyses and computational models

As a way forward, Green et al. (2019) recommended well-powered studies with large numbers of participants. In a similar vein, and focusing on the *n*-back-task training, Pergher et al. (2020) proposed large-scale studies isolating promising features. We believe that such an atheoretical approach is unlikely to succeed. There is an indefinite space of possible interventions (e.g., varying the type of training task, the cover story used in a game, the perceptual features of the material, the pace of presentation, ad infinitum), which means that searching this space blindly and nearly randomly would require a prohibitive amount of time. Strong theoretical constraints are needed to narrow down the search space.

There is thus an urgent need to understand which cognitive mechanisms might lead to cognitive transfer. As we showed above in the section on meta-analysis, the available evidence shows that the real effect size of cognitive training on far transfer is zero. Prima facie, this outcome indicates that theories based on general mechanisms, such as brain plasticity (Karbach & Schubert, 2013), primitive elements (Taatgen, 2013), and learning to learn (Bavelier et al., 2012), are incorrect when it comes to far transfer. We reach this conclusion by a simple application of modus tollens: (a) Theories based on general mechanisms such as brain plasticity, primitive elements, and learning to learn predict far transfer. (b) The empirical evidence shows that there is no far transfer. Therefore, (c) theories based on general mechanisms such as brain plasticity, primitive elements, and learning to learn are incorrect.

Thus, if one believes that cognitive training leads to cognitive enhancement—most likely limited to near transfer—one has to come up with other theoretical mechanisms than those currently available in the field. We recommend two approaches to identify such mechanisms, which we believe should be implemented before large-scale randomized controlled trials are carried out.

##### Fine analyses of the processes in play

The first approach is to use experimental methods enabling the identification of cognitive mechanisms. Cognitive psychology has a long history of refining such methods, and we limit ourselves to just a few pointers. A useful source of information consists in collecting fine-grained data, such as eye movements, responses times, and even mouse location and mouse clicks. Together with hypotheses about the processes carried out by participants, these data make it possible to rule out some mechanisms while making others more plausible. Another method is to design experiments that specifically test some theoretical mechanisms. Note that this goes beyond establishing that a cognitive intervention leads to some benefits compared with a control group. In addition, the aim is to understand the specific mechanisms that lead to this superiority.

It is highly likely that the strategies used by the participants play a role in the training, pretests, and posttests used in cognitive-training research (Sala & Gobet, 2019; Shipstead et al., 2012; von Bastian & Oberauer, 2014). It is essential to understand these strategies and the extent to which they differ between participants. Are they linked to a specific task or a family of tasks (near transfer), or are they general across many different tasks (far transfer)? If it turns out that such general strategies exist, can they be taught? What do they tell researchers about brain plasticity and changing basic cognitive abilities such as general intelligence?

Two studies that investigated the effects of strategies are mentioned here. Laine et al. (2018) found that instructing participants to employ a visualization strategy when performing *n*-back training improved performance. In a replication and extension of this study, Forsberg et al. (2020) found that the taught visualization strategy improved some of the performance measures in novel *n*-back tasks. However, older adults benefited less, and there was no improvement in WM tasks structurally different from *n*-back tasks. In the uninstructed participants, *n*-back performance correlated with the type of spontaneous strategies and their level of detail. The types of strategies also differed as a function of age.

A final useful approach is to carry out a detailed task analysis (e.g., Militello & Hutton, 1998) of the activities involved in a specific regimen of cognitive training and in the pretests and posttests used. What are the overlapping components? What are the critical components and those that are not likely to matter in understanding cognitive training? These components can be related to information about eye movements, response times, and strategies and can be used to inspire new experiments. The study carried out by Baniqued et al. (2013) provides a nice example of this approach. Using task analysis, they categorized 20 web-based casual video games into four groups (WM, reasoning, attention, and perceptual speed). They found that performance in the WM and reasoning games was strongly associated with memory and fluid-intelligence abilities, measured by a battery of cognitive tasks.

##### Cognitive modeling as a method

The second approach we propose consists of developing computational models of the postulated mechanisms, which of course should be consistent with what is known generally about human cognition (for a similar argument, see Smid et al., 2020). To enable an understanding of the underlying mechanisms and be useful in developing cognitive-training regimens, the models should be in a position to simulate not only the tasks used as pretests and posttests but also the training tasks. This is what Taatgen’s (2013) model is doing: It first simulates improvement in a complex verbal WM task over 20 training sessions and then simulates how WM training reduces interference in a Stroop task compared with a control group. (We would, of course, query whether this far-transfer effect is genuine.) By contrast, Green, Pouget, & Bavelier’s (2010) neural-network and diffusion-to-bound models simulate the transfer tasks (a visual-motion-direction discrimination task and an auditory-tone-location discrimination task) but do not simulate the training task with action video-game playing. Ideally, a model of the effect of an action video game should simulate actual training (e.g., by playing *Call of Duty 2*), processing the actual stimuli involved in the game. To our knowledge, no such model exists. Note that given the current developments in technology, modeling such a training task is not unrealistic.

The models should also be able to explain data at a micro level, including eye movements and verbal protocols (to capture strategies). There is also a need for the models to use exactly the same stimuli as those used in the human experiments. For example, the chunk hierarchy and retrieval structures model of chess expertise (De Groot et al., 1996; Gobet & Simon, 2000) receives as learning input the kind of board positions that players are likely to meet in their practice. When simulating experiments, the same stimuli are used as those employed with human players, and close comparison is made between predicted and actual behavior along a number of dimensions, including percentage of correct responses, number and type of errors, and eye movements. In the field of cognitive training, Taatgen’s (2013) model is a good example of the proper level of granularity for understanding far transfer. Note that, ideally, the models should be able to predict possible confounds and how modifications to the design of training would circumvent them. Indeed, we recommend that considerable resources be invested in this direction of research with the aim of testing interventions in silico before testing them in vivo (Gobet, 2005). Only those interventions that lead to benefits in simulations should be tested in trials with human participants. In addition to embodying sound principles of theory development and testing, such an approach would also lead to considerable savings of research money in the medium and long terms.

### Searching for small effects

Green et al. (2019, p. 20) recognized the possibility that large effects are unlikely and that one should be content with small effects. They are also open to the possibility of using unspecific effects, such as expectation effects. It is known that many educational interventions bring a modest effect (Hattie, 2009), and thus, the question arises as to whether cognitive-training interventions are more beneficial than alternative ones. We argue that many other interventions are cheaper and/or have specific benefits when they directly match educational goals. For example, games related to mathematics are more likely to improve one’s mathematical knowledge and skills than *n*-back tasks and can be cheaper and more fun.

If cognitive training leads only to small and unspecific effects, one faces two implications, one practical and one theoretical. Practically, the search for effective training features has to operate blindly, which is very inefficient. This is because current leading theories in the field are incorrect, as noted above, and thus there is no theoretical guidance. Thus, effectiveness studies are unlikely to yield positive results. Theoretically, if the effectiveness of training depends on small details of training and pre/post measures, then the prospects of generalization beyond specific tasks are slim to null. This is unsatisfactory scientifically because science progresses by uncovering general laws and finding order in apparent chaos (e.g., the state of chemistry before and after Mendeleev’s discovery of the periodic table of elements).

A straightforward explanation can be proposed for the pattern of results found in our meta-analyses with respect to far transfer—small to zero effect sizes, low or null true between-studies variance. Positive effect sizes are just what can be expected by chance, features of design (i.e., active vs. passive control groups), regression to the mean, and sometimes publication bias. (If you believe that explanations based on chance are not plausible, consider Galton’s board: It perfectly illustrates how a large number of small effects can lead to a normal distribution. Likewise, in cognitive training, multiple variables and mechanisms lead to some experiments having a positive effect, others a negative effect, with most experiments centered around the mean of the distribution.) Thus, the search for robust and replicable effects is unlikely to be successful.

Note that the issue with cognitive training is not the lack of replications and the lack of reproducibility, which plague large swathes of psychology: The main results have been replicated often and form a highly coherent pattern when results are put together in (meta-)meta-analyses. Pace Pergher et al. (2020), we do not believe that variability of methods is an issue. On the contrary, the main outcomes are robust to experimental variations. Indeed, results obtained with many different training and evaluation methods converge (small-to-zero effect sizes and low true heterogeneity) and thus satisfy a fundamental principle in scientific research: the principle of triangulation (Mathison, 1988).

### Funding agencies

Although Green et al.’s (2019) article is explicitly about methodology, it does make recommendations for funding agencies and lobbies for more funding: “We feel strongly that an increase in funding to accommodate best practice studies is of the utmost importance” (p. 17). On the one hand, this move is consistent with the aims of their article in that several of the suggested practices, such as using large samples and performing studies that would last for several years, would require substantial amounts of money to be carried out. On the other hand, lobbying for an increase in funding is made without any reference to results showing that cognitive training might not provide the hoped-for benefits. The authors only briefly discussed the inconsistent evidence for cognitive training, concluding that “our goal here is not to adjudicate between these various positions or to rehash prior debates” (p. 3). However, in general, rational decisions about funding require an objective evaluation of the state of the research. Obviously, if the research is about developing methods for cognitive enhancement, funders must take into consideration the extent to which the empirical evidence supports the hypothesis that the proposed methods provide domain-general cognitive benefits. As we showed in the “Meta-Analytical Evidence” section, there is little to null support for this hypothesis. Thus, our advice for funders is to base their decisions on the available empirical evidence and on the conclusions reached by meta-analyses.

## The Broader View

As discussed earlier, our meta-analyses clearly show that cognitive training does not lead to any far transfer in any of the cognitive-training domains that have been studied. In addition, using second-order meta-analysis made it possible to show that the between-meta-analyses true variance is due to second-order sampling error and thus that the lack of far transfer generalizes to different populations and different tasks. Taking a broader view suggests that our conclusions are not surprising and are consistent with previous research. In fact, they were predictable. Over the years, it has been difficult to document far transfer in experiments (Singley & Anderson, 1989; Thorndike & Woodworth, 1901), industrial psychology (Baldwin & Ford, 1988), education (Gurtner et al., 1990), and research on analogy (Gick & Holyoak, 1983), intelligence (Detterman, 1993), and expertise (Bilalić et al., 2009). Indeed, theories of expertise emphasize that learning is domain-specific (Ericsson & Charness, 1994; Gobet & Simon, 1996; Simon & Chase, 1973). When putting this substantial set of empirical evidence together, we believe that it is possible to conclude that the lack of training-induced far transfer is an invariant of human cognition (Sala & Gobet, 2019).

Obviously, this conclusion conflicts with the optimism displayed in the field of cognitive training, as exemplified by Green et al.’s (2019) article discussed above. However, it is in line with skepticism recently expressed about cognitive training (Moreau, 2021; Moreau et al., 2019; Simons et al., 2016). It also raises the following critical epistemological question: Given that the overall evidence in the field of cognitive training strongly suggests that the postulated far-transfer effects do not exist, and thus the probability of finding such effects in future research is very low, should one conclude that the reasonable course of action is to stop performing cognitive-training research on far transfer?

We believe that the answer to this question is “yes.” Given the clear-cut empirical evidence, the discussion about methodological concerns is irrelevant, and the issue becomes searching for other cognitive-enhancement methods. However, although the hope of finding far-transfer effects is tenuous, the available evidence clearly supports the presence of near-transfer effects. In many cases, near-transfer effects are useful (e.g., with respect to older adults’ memory), and developing effective methods for improving near transfer is a valuable—and importantly, realistic—avenue for further research.

## Transparency

*Action Editor:* Laura A. King

*Editor:* Laura A. King

*Author Contributions*

F. Gobet conceived the idea of the article. F. Gobet and G. Sala wrote the manuscript. Both authors approved the final manuscript for submission.

## Notes

1. Because our definition focuses on cognitive tasks, it does not include mostly physical activities, such as sport. In addition, note that the term “cognitive training” is also used in a different line of research in which the interest is in testing the limits of cognitive plasticity in ageing, for example by training younger and older participants to use mnemonics (e.g., Kliegl et al., 1989).

2. For a broader conceptualization of transfer, see Barnett and Ceci (2002) and Klahr and Chen (2011).

3. When a random-effect meta-analysis is performed, the effect sizes are weighted on the inverse of the sum of their sampling error and the between-studies true variance (τ^2^).

4. The article listed in this section contain extensive discussions of the meta-analyses carried out by other authors.

5. Au and colleagues (2020) violated the assumption of statistical independence by grouping meta-analyses with overlapping samples into a number of clusters. Although the clusters’ overall effect sizes were statistically independent to each other, these effect sizes and their sampling error variances were incorrectly calculated as a result of the aforementioned violation.
